# Microevolution of *Vibrio parahaemolyticus* Isolated from Clinical, Acute Hepatopancreatic Necrosis Disease Infecting Shrimps, and Aquatic Production in China

**DOI:** 10.1264/jsme2.ME19095

**Published:** 2020-03-20

**Authors:** Yi Lu, Lulu Yang, Jing Meng, Yong Zhao, Yishan Song, Yongheng Zhu, Jie Ou, Yingjie Pan, Haiquan Liu

**Affiliations:** 1 College of Food Science and Technology, Shanghai Ocean University, Shanghai 201306; 2 Shanghai First Maternity and Infant Hospital, Tongji University School of Medicine, Shanghai 201204; 3 Shanghai Engineering Research Center of Aquatic-Product Processing & Preservation, Shanghai 201306; 4 Laboratory of Quality & Safety Risk Assessment for Aquatic Product on Storage and Preservation (Shanghai), Ministry of Agriculture, Shanghai 201306; 5 Engineering Research Center of Food Thermal-processing Technology, Shanghai Ocean University, Shanghai 201306

**Keywords:** *Vibrio parahaemolyticus*, microevolution, MLST

## Abstract

*Vibrio parahaemolyticus* is the leading cause of bacteria-associated foodborne diarrheal diseases and specifically causes early mortality syndrome (EMS), which is technically known as acute hepatopancreatic necrosis disease (AHPND), a serious threat to shrimp aquaculture. To investigate the genetic and evolutionary relationships of *V. parahaemolyticus* in China, 184 isolates from clinical samples (VPC, *n*=40), AHPND-infected shrimp (VPE, *n*=10), and various aquatic production sources (VPF, *n*=134) were collected and evaluated by a multilocus sequence analysis (MLST). Furthermore, the presence of potential virulence factors (*tlh*, *tdh*, and *trh*) and single nucleotide polymorphisms (SNPs) in *V. parahaemolyticus* isolates was assessed using genomic sequencing. Analyses of virulence factors revealed that the majority of VPC isolates (97.5%) possessed the *tdh* and/or *trh* genes, while most of the VPF isolates (83.58%) did not encode hemolysin genes. Therefore, we hypothesized that the environment is a potential reservoir that promotes horizontal DNA transfer, which drives evolutionary change that, in turn, leads to the emergence of novel, potentially pathogenic strains. Phylogenetic analyses identified VPF-112 as a non-pathogenic maternal strain isolated from aquatic products and showed that it had a relatively high evolutionary status. All VPE strains and some VPC strains were grouped into several small subgroups and evenly distributed on phylogenetic trees. Anthropogenic activities and environmental selective pressure may be important factors influencing the process of transforming strains from non-pathogenic to pathogenic bacteria.

*Vibrio parahaemolyticus* is a halophilic marine microorganism that causes foodborne infections, such as acute gastroenteritis, when consumed through raw or partially cooked seafood (Zhang and Orth, 2013). It is currently the leading cause of seafood-borne diseases, and is gradually impacting global public health (Drake *et al.*, 2010; Letchumanan *et al.*, 2014). It is widely disseminated in estuarine areas, with epidemic outbreaks typically occurring in coastal countries and tropical and temperate regions (Alam *et al.*, 2002; Gil *et al.*, 2007; Ansaruzzaman *et al.*, 2008; Castillo *et al.*, 2018). Seafood-associated infections by *V. parahaemolyticus* were initially reported in Japan in the early 1950s (Ansaruzzaman *et al.*, 2008), and their incidence has increased not only in coastal countries in which the temperature of seawater is warm, but also in cold northern regions (Velazquez-Roman *et al.*, 2014; Soto-Rodriguez *et al.*, 2015; Xu *et al.*, 2016). Thirty-six *V. parahaemolyticus* outbreaks occurred in the Guangdong province of China between 2008 and 2010 (Ma *et al.*, 2014), while 71 outbreaks caused by *V. parahaemolyticus* were reported in the Zhejiang province of China between 2010 and 2014, resulting in 933 infections and 117 hospitalizations (Jiang *et al.*, 2017). Recent surveillance data (Wong *et al.*, 2000; Pazhani *et al.*, 2014) revealed that surveillance and epidemiological investigations on the incidence of infections by this pathogen have reduced the burden of disease. Therefore, the further accumulation of epidemiological data and clarification of the microevolutionary relationship of *V. parahaemolyticus* are important for the development of strategies to reduce the burden of disease in China.

The main virulence factors of *V. parahaemolyticus* are thermostable direct hemolysin (TDH) and TDH-related hemolysin (TRH) (Castillo *et al.*, 2018), which are encoded by the *tdh* and *trh* genes, respectively (Ceccarelli *et al.*, 2013; Raghunath, 2014). *V. parahaemolyticus* isolates containing *tdh* and/or *trh* are generally regarded as a public health threat (Broberg *et al.*, 2011; Ceccarelli *et al.*, 2013; Zhang and Orth, 2013) and food contaminated by these strains have the potential to cause human illness (Pazhani *et al.*, 2014; Xie *et al.*, 2017). *tdh* and *trh* in *V. parahaemolyticus* isolates may be easily and cost-effectively detected using PCR-based methods (West *et al.*, 2013). *V. parahaemolyticus* is a human and shrimp pathogen. Acute hepatopancreatic necrosis disease (AHPND) is caused by specific strains of *V. parahaemolyticus* that harbor a plasmid encoding toxin genes (Gomez-Gil *et al.*, 2014; Kondo *et al.*, 2014; Koiwai *et al.*, 2015). Previous outbreaks of AHPND resulted in large economic losses in the global shrimp farming industry as well as a series of socioeconomic issues, particularly in Asia, Mexico, and America (Flegel, 2012; Zhang *et al.*, 2012; Tran *et al.*, 2013; Gomez-Gil *et al.*, 2014; Soto-Rodriguez *et al.*, 2015; Devadas *et al.*, 2018). This disease first appeared in China in 2009 and caused significant economic losses (Hong *et al.*, 2016; Fu *et al.*, 2018).

Shanghai, as a port city and the economic center of China, imports and exports significant volumes of aquatic products. Several cases of *V. parahaemolyticus* infections were attributed to the wide variety of seafood restaurants with diverse eating habits (Guo *et al.*, 2013; Yu *et al.*, 2016), which represents a potential threat for public health. The prevalence of *V. parahaemolyticus* infections and diversity in aquatic products and the origins of clinical isolates have been widely reported (Jones *et al.*, 2012; Ludeke *et al.*, 2014; He *et al.*, 2016; Lou *et al.*, 2016; Xu *et al.*, 2016; Yu *et al.*, 2016; Li *et al.*, 2017; Xie *et al.*, 2017); however, limited information is currently available on the microevolution of *V. parahaemolyticus* isolated from aquatic products, clinical isolates, and AHPND-infected shrimp. In the present study, we used a multilocus sequence analysis (MLST) to investigate the genetic characterization and population structure of *V. parahaemolyticus* isolated from aquatic products, infected shrimp, and clinical epidemiological samples. The major purposes of this study were to elucidate the microevolution relationship of *V. parahaemolyticus* isolates from Shanghai and Guangdong province by clarifying their virulence, genetic diversity, and the presence of single nucleotide polymorphisms (SNPs). We intend to obtain credible data through the continuous monitoring of *V. parahaemolyticus* and show a phylogenetic correlation between pathogens and non-pathogens in order to improve the management and treatment of foodborne infections and effectively reduce economic losses in the seafood industry.

## Materials and Methods

### *V. parahaemolyticus* strain information and growth conditions

One hundred and eighty-four *V. parahaemolyticus* isolates were collected from three different sources in Shanghai and Guangdong province. Clinical isolates (VPC, *n*=40) were recovered from patients who presented with acute diarrhea to the gastroenteritis outpatient clinics of Shanghai hospitals. Environmental isolates (VPF, *n*=134) were recovered from Shanghai aquatic product markets. AHPND isolates (VPE, *n*=10) were collected from shrimp infected with AHPND in Guangdong province. Details on these isolates are shown in Table 1.

All of the *V. parahaemolyticus* isolates used in the present study were stored in 5-mL glycerol tubes with 25% glycerol at –80°C. *V. parahaemolyticus* strains were inoculated into 10‍ ‍mL tryptic soy broth (TSB; Beijing Land Bridge Technology) with 3% NaCl and grown at 37°C for 12–16 h with shaking at 180 rpm. A few colonies were transferred for plate streaking to thiosulfate-citrate-bile salts-sucrose agar culture medium (TCBS; Beijing Land Bridge Technology)-selective plates, and cultured in an inverted position at 37°C for 18–24 h. A single green colony on the TCBS plate was selected with a sterile inoculating loop and transferred into 10‍ ‍mL of 3% NaCl TSB at 37°C for 12–16 h with shaking at 180 rpm, and the last step for the preparation of DNA extraction was repeated.

### DNA extraction

The total DNA of the 184 *V. parahaemolyticus* isolates was extracted using the TIANamp Bacteria DNA isolation Kit (Tiangen Biotech Beijing) according to the recommendations for commercial protocols, and DNA samples were stored at –20°C.

### Detection of virulence-associated genes

The presence of the species-specific gene *tlh* was examined in all 184 *V. parahaemolyticus* isolates using a polymerase chain reaction (PCR). The primers used in the present study were *tlh*-F (5′-AAA GCG GAT TAT GCA GAA GCA CTG-3′) and *tlh*-R (5′-GCT ACT TTC TAG CAT TTT CTC TGC-3′). The virulence genes *tdh* (West *et al.*, 2013) and *trh* (Yoh *et al.*, 2010) of *V. parahaemolyticus* strains were also detected by PCR. The oligonucleotide primers used in the present study were synthesized by Sangon Biotech. The reference strains were *V. parahaemolyticus* ATCC17802 (*trh^+^*) and ATCC33847 (*tdh^+^*), and the negative control was distilled water.

The PCR sequencing reactions and PCR assay used were described previously (Li *et al.*, 2017). Briefly, PCR sequencing reactions were performed in 50‍ ‍μL of reaction mixture, and each reaction mixture consisted of 25‍ ‍μL of PCR Mix (Sangon Biotech), 3‍ ‍μL of a DNA template, 20‍ ‍μL of double-distilled water, and 1‍ ‍μL of each primer. PCR assays (Supplementary Table S4) were performed using the following amplification parameters: an initial denaturation step at 94°C for 3‍ ‍min, 25 cycles of denaturation at 94°C for 1‍ ‍min, annealing at 62–66°C for 1‍ ‍min, and extension at 72°C for 2‍ ‍min, with a final extension at 72°C for 3‍ ‍min. We then used agarose gel electrophoresis to analyze PCR products.

### MLST and nucleotide and population structure analyses

We selected seven housekeeping genes from chromosomal DNA: *dna*E (596 bp), *gyr*B (629 bp), *rec*A (773 bp), *dtd*S (497‍ ‍bp), *pnt*A (470 bp), *pyr*C (553 bp), and *tna*A (463 bp) (Supplementary Table S1), to examine *V. parahaemolyticus* genetic traits based on MLST (Maiden *et al.*, 2013). PCR primers were aligned using DNAMAN sequence analysis software and sequenced by Sangon Biotech (Sangon Biotech). The forward and reverse primers of seven housekeeping gene allele sequences were aligned and edited by DNAMAN and generated into a consensus sequence. Each consensus sequence of the isolates was submitted to the *V. parahaemolyticus* MLST website (http://pubmlst.org/vparahaemolyticus) to assign allele numbers and identify sequence types (STs). New allele sequences and novel ST profiles that did not match any pre-existing sequence data within the database were submitted to the database curator, who assigned it as a new allele and ST. After analyzing the genetic traits of *V. parahaemolyticus* isolates, the Global optimal eBURST (goeBURST) full MST algorithm was used to compare the PubMLST database in order to highlight the potential relationships of different strain isolates. The genetic information and evolutionary characteristics of all strains were considered. Strain relationships were then analyzed using the PHYLOViZ program to identify potential CCs. Nucleotide sequence analyses were conducted with MEGA. The distribution of SNPs among the 184 concatenated sequences of *V. parahaemolyticus* was assessed in relation to the complete sequence of *V. parahaemolyticus* strain ATCC 17802.

## Results

### Diversity of STs

**Fig. 1.**
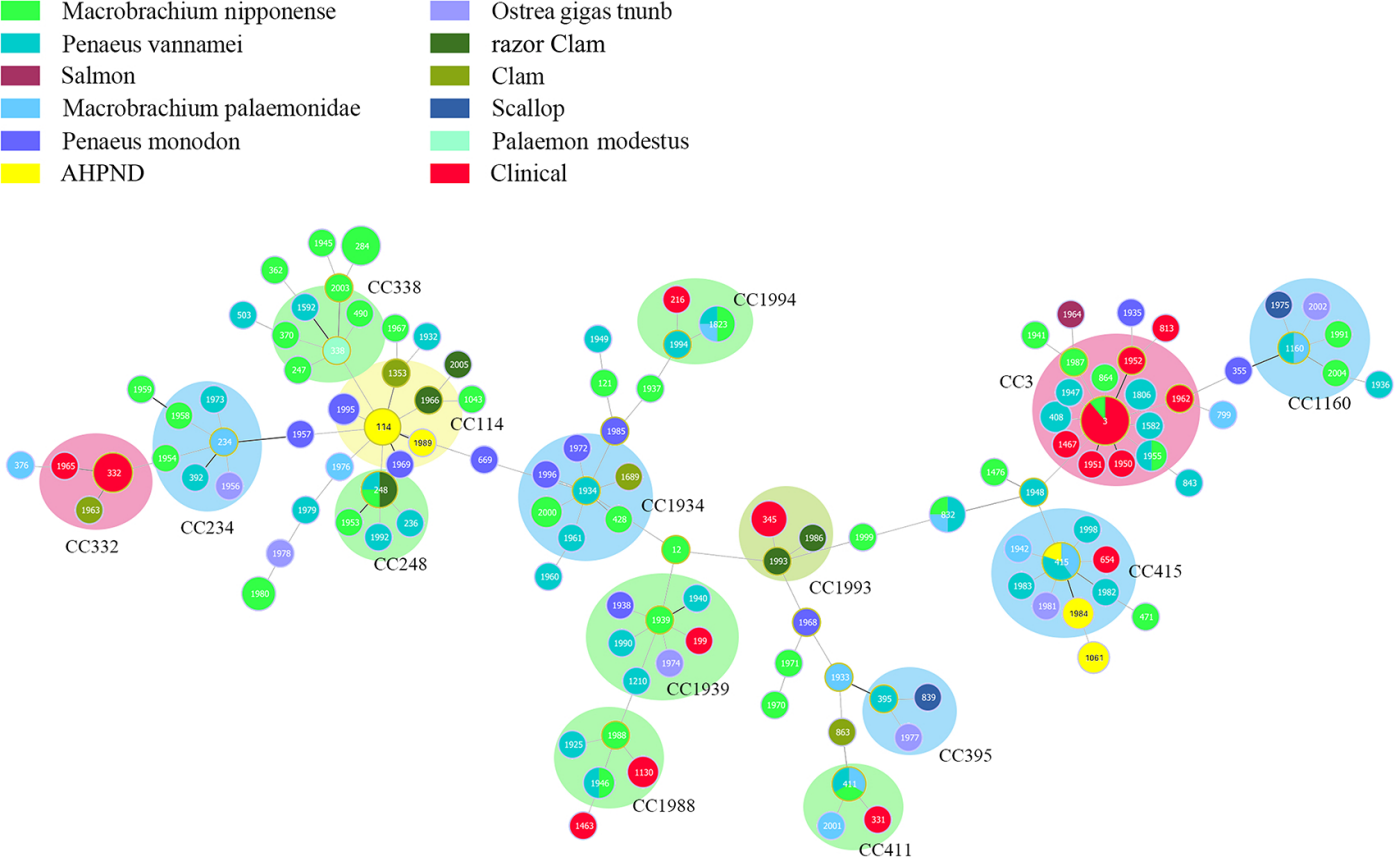
goeBURST full MST analysis of 184 STs of *V. parahaemolyticus*. Each circle represents a ST and the size of the circle reflects the frequency of each ST in the dataset. The number of different alleles is presented between STs connected via a line. Each shaded area represents a unique clone complex (CC).

The source, year isolated, presence of a species-specific gene (*tlh*) and potential virulence factors (*tdh* and *trh*), STs, and allelic profiles of the 184 *V. parahaemolyticus* isolates are shown in Table 1. The outcomes of MLST classified the 184 *V. parahaemolyticus* isolates into 124 STs (Fig. 1), 80 of which were novel. A total of 134 isolates were detected in the VPF group, including 105 different and 71 new STs. Forty isolates were extracted from the VPC group and 10 from the VPE group, which contained 16 and 5 distinct STs with 7 and 2 novel STs, respectively. Sequence variations were significantly higher in VPF isolates (105 STs) than in the other two groups (21 STs). This high number of different STs indicated the potential of the environment as a reservoir.

Thirty-one STs included more than one isolate each: ST3 included 18; ST284 and ST332 each had 6; ST415 included 5; STs 248, 345, 832, and 1823 each had 4; ST411 and ST1980 each included 3; and STs 408, 1061, 1130, 1160, 1806, 1946, 1955, 1984, 1995, and 2000 each had 2. In addition, 9 STs (ST3, ST248, ST411, ST415, ST832, ST1160, ST1823, ST1946, and ST1955) presented mixed colors in the corresponding circle (Fig. 1). A mixed origin was observed in two STs, which were ST3 (VPF and VPC) and ST415 (VPF and VPE), indicating the importance of gene sequences from other species.

### Identification of clonal complexes

The goeBURST full MST algorithm used in the present study categorized all STs into 15 clone complexes (CC), 7 of which were novel (CC332, CC1160, CC1934, CC1939, CC1988, CC1993, and CC1994), revealing high genetic diversity (Fig. 1). Seven CCs (CC3, CC332, CC411, CC1939, CC1988, CC1993, and CC1994) were identified in VPF and VPC mixed isolates, while only one (CC114) was detected in VPF and VPE mixed isolates. The other six CCs (CC234, CC248, CC338, CC395, CC1160, and CC1934) were identified in environmental isolates only. Among these, CC3 was the largest clone complex with frequent isolates and STs, including 33 isolates with VPC (63.64%) and VPF (36.36%), and 13 STs, 10 of which were novel. CC415 was identified as the only ST that caused a wide range of infections in three groups with five species: VPE (AHPND-infected shrimp), VPC (clinical samples), some VPF (mainly including *Penaeus vannamei*, Macrobrachium: Palaemonidae, and Ostrea gigas tnunb). CC114 was the only CC comprising two groups recovered from VPE (AHPND-infected shrimp) and VPF (*Penaeus monodon*, Clam, and Razor Clam). The mixed origin of CCs suggested intra- and interspecies recombination events involving the three groups of isolates.

### Distribution of virulence-associated genes

In the present study, different hemolysin gene profiles were observed among the VPF, VPC, and VPE isolates. The majority of isolated VPF strains (83.58%) did not encode hemolysin genes, while the remaining isolates carried the *tdh* gene (11.19%), and 5.22% of isolates harbored the *trh* gene. In the case of VPE, all VPE isolates were non-pathogenic strains (*tlh^+^/tdh^–^/trh^–^*). However, the majority of VPC strains (80.00%) possessed only the *tdh* gene, whereas the isolates VPC-06, VPC-09, VPC-19, and VPC-27 (10.00%) contained only the *trh* gene. Only one strain, VPC-34 (2.50%) did not encode hemolysin genes, while VPC-18, VPC-23, and VPC-40 (7.50%) were positive for the *tdh* and *tlh* genes. These three strains were evenly distributed rather than being clustered together on the phylogenetic tree, indicating that they did not evolve from the same maternal strains. The majority of VPC strains clustered together in lineage A, while other VPC strains, which were closely related to VPF and VPE strains, clustered in several subgroups that were evenly distributed on the phylogenetic tree.

### *V. parahaemolyticus* showed a differential seasonal distribution

**Fig. 2.**
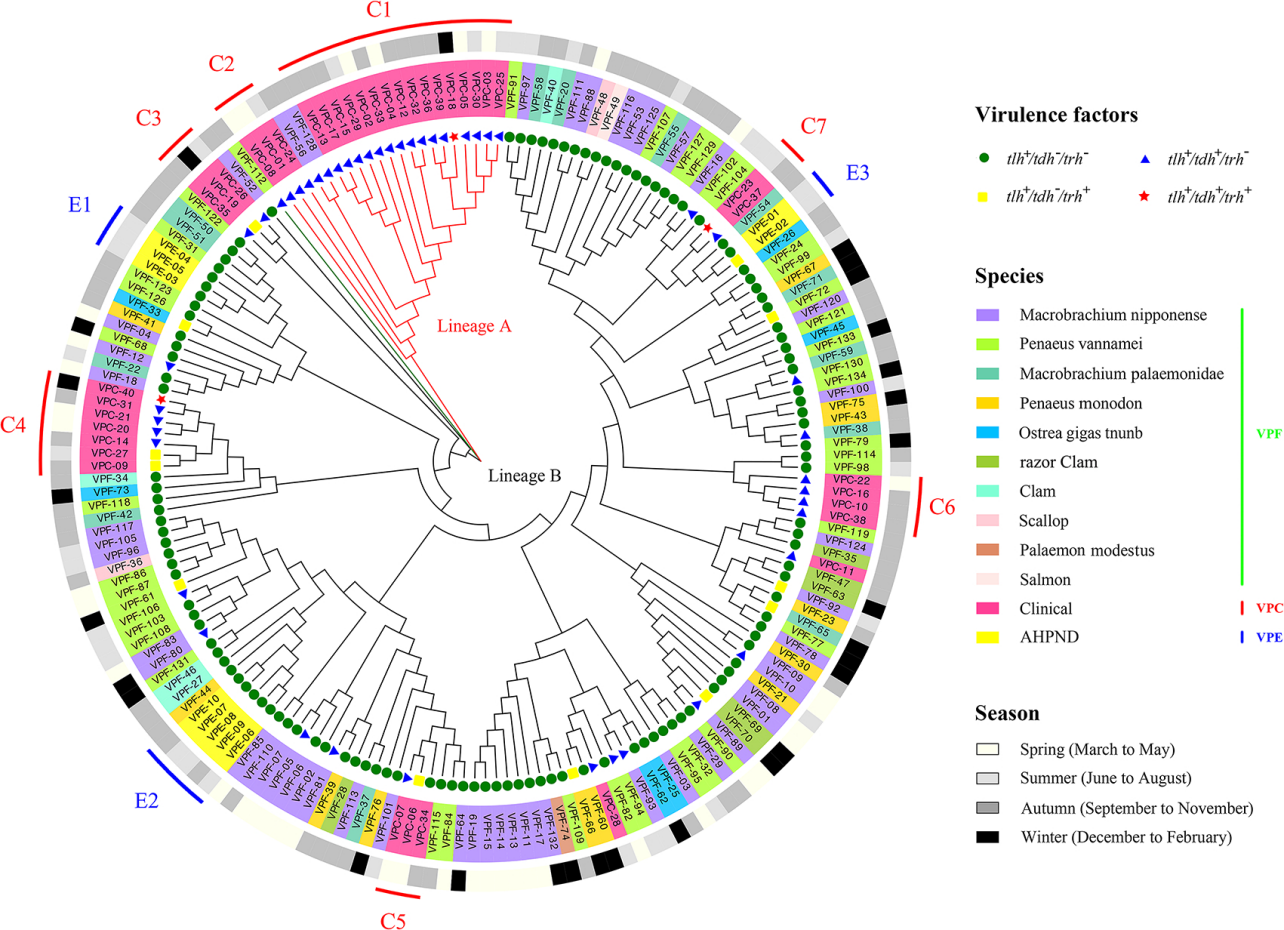
Phylogenetic tree of 184 *V. parahaemolyticus* isolates analyzed in this study. The colored shapes refer to virulence factors. The inner ring shows the different biological sources of the strains. The seasons of sampling are presented in the outer ring.

The effects of seasonality on strain diversity were also examined in the present study, with the sampling time being presented on the outer ring (Fig. 2) as per the seasons in Shanghai. In the VPF group, the specimen numbers for the four seasons were 29 (spring), 21 (summer), 59 (autumn), and 25 (winter), with the number of pathogenic strains for each season being 5, 5, 6, and 6, respectively. Although the sample size in autumn was approximately 3-fold higher than those of the other seasons, the number of pathogens was similar. However, the evolution status of the strains in warm seasons (summer-autumn) was more primitive than those in cold seasons (winter-spring). In the VPE group, all strains were separated in warm seasons; in the VPC group, more than 50% of the strains (65.00%) were isolated during warm seasons, which was approximately 2-fold more than those in the cold season. Only three isolates, VPC-18, VPC-23, and VPC-40, were positive for the *tdh* and *tlh* genes, and originated from human clinical samples. Two out of the three isolates were collected in winter, while the other was collected in summer. Therefore, in the VPC and VPE groups, the greater strain diversity observed in warm season collections than in cold season collections indicated that when water temperature increased, *V. parahaemolyticus* isolates become more diverse and the cold-tolerant subpopulation was gradually replaced.

### Phylogenetic analysis

A neighbor-joining tree representing the concatenated sequences of the seven housekeeping gene fragments of 184 isolates is shown in Fig. 2. The phylogenetic tree consisted of two major lineages, A (red branch color) and B (black branch color). The isolate VPF-112 (green branch color in), with a relatively high evolutionary status, did not cluster in lineage A or B, and was separated from the other isolates by a relatively large genetic distance. This result showed that lineage A only included two distinct strain populations, clinical samples (*n*=19) and *Macrobrachium nipponense* (*n*=2), while lineage B was further subdivided into 13 closely related clades. The isolates recovered from clinical samples, AHPND-infected shrimp, and various aquatic production sources were highly diverse and distributed widely throughout the tree.

Thirty-eight clinical isolates were divided into 7 subgroups (C1–C7, red line), except for 2 clinical isolates (VPC-11 and VPC-28) that were separately distributed. C1–C3 clustered together showing a close phylogenetic relationship, while C4–C7 were distributed evenly in the tree. Lineage A included C1, C2, and two VPF isolates. Consistent with the current MLST scheme, these strains were all classified into a clonal complex (CC3), except for VPC-04 and VPC-25, which were novel ST1950 and ST1951 respectively. Apart from the *gyrB* of VPC-04 and *pntA* of VPC-25, the six remaining loci of these 2 isolates were identical to those belonging to CC3 (ST3). This result suggests that these 2 isolates belonged to CC3 and were single sequence variants (SLV) on the locus of their ancestral type (ST3).

### Nucleotide diversity of *V. parahaemolyticus*

**Fig. 3.**
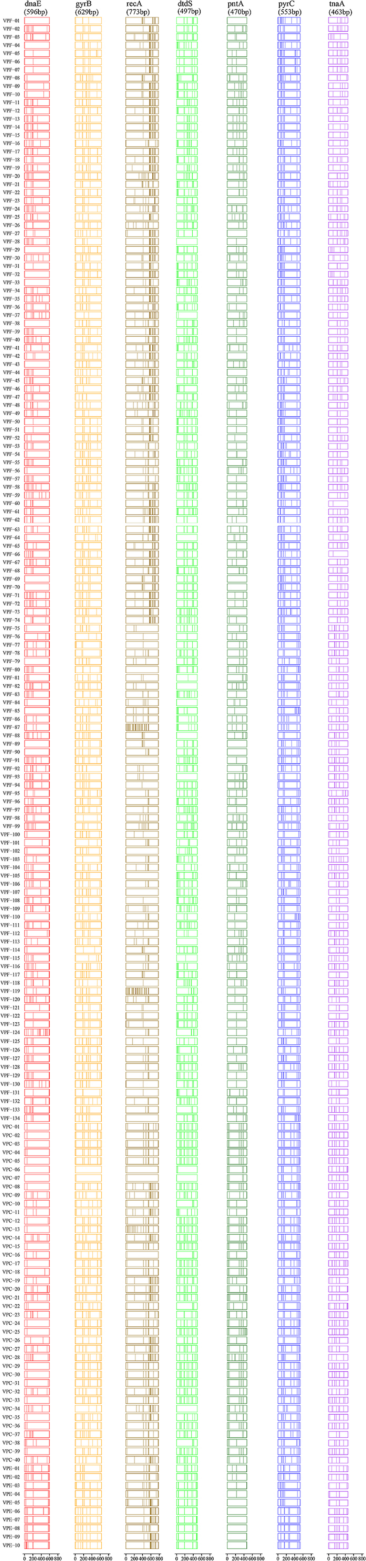
Distribution of polymorphic nucleotide sites among 184 concatenated sequences of *V. parahaemolyticus*. The seven housekeeping genes of each strain correspond to one of the seven bands, each vertical line in the band represents a mutation site (relative to ATCC 17802), and the density of the vertical line in the band clearly reflects the relative position of the mutation site. The demarcation and nucleotide lengths of the seven genes are indicated along the bottom scale.

The SNPs of seven housekeeping gene comparisons were used to elucidate the relationships and SNPs of strains with different origins (Fig. 3). The mutation sites in *dnaE* and *pyr*C mainly appeared at the beginning of the band and those of *rec*A are mainly concentrated at the rear end of the band, while the mutation sites of the four other genes were evenly distributed throughout the band. The total SNP ratios of the seven housekeeping genes were 0.92% (*dna*E), 1.43% (*gyr*B), 1.47% (*rec*A), 1.77% (*dtd*S), 0.71% (*pnt*A), 1.34% (*pyr*C), and 1.26% (*tna*A). The highest and lowest SNP ratios were observed in *dtd*S and *pnt*A, respectively, and there was no correlation between the SNP ratio and gene fragment length. Moreover, the SNP ratios of the three groups were estimated to be 1.24% (VPF), 1.41% (VPC), and 1.43% (VPE), while the SNP ratios of the VPC and VPE groups and clinical and diseased shrimp origins were significantly higher than that of the VPF group. The *rec*A gene is considered to possess the largest number of mutation sites, while, as previously described, the SNP ratio of *dtd*S was the highest among the seven housekeeping genes; therefore, both of them significantly influence the population structure and apparent phylogenetic relationships of *V. parahaemolyticus*.

The isolate VPF-112 (green branch color) did not cluster in lineage A or B (Fig. 2), showing evolutionary conservation. We initially suspected that the marked variation in VPF-112 may due to frequent recombination or mutations. However, the results shown in Fig. 2 and 3 revealed that the numbers of mutation sites in the seven housekeeping genes of VPF-112 were only 4 (*dna*E), 8 (*gyr*B), 2 (*rec*A), 3 (*dtd*S), 5 (*pnt*A), 6 (*pyr*C), and 7 (*tna*A), which were markedly lower than the average number of mutations in each housekeeping gene: (VPC and VPE) 4 (*dna*E), 10 (*gyr*B), 12 (*rec*A), 9 (*dtd*S), 6 (*pnt*A), 8 (*pyr*C), and 8 (*tna*A). This result indicates that strains with a higher SNP ratio were more likely to cause illness in humans or disease in shrimp.

## Discussion

In the present study, all 184 *V. parahaemolyticus* isolates were classified into 124 STs, of which 80 were novel. The results obtained also showed that STs varied, even when *V. parahaemolyticus* was recovered from a single city, and most of the STs in the environment were non-pathogenic, revealing the high diversity of *V. parahaemolyticus*. ST3 is generally known as a pandemic complex that has the potential to be a significant public health risk (Turner *et al.*, 2013). Two VPF (environmental isolates) represent ST3, and, thus, the isolates that are members of the same ST and phylogenetic clade are capable of survival in cooperation with several types of species. Therefore, the continuous monitoring of *V. parahaemolyticus* is of importance.

Previous studies reported that the majority of clinical *V. parahaemolyticus* isolates contained the *tdh* and/or *trh* gene and have the potential to become a major public health threat (Zhang *et al.*, 2017). However, some environmental *V. parahaemolyticus* isolates may also possess the *tdh* and/or *trh* gene (Xu *et al.*, 2016); therefore, the relationship between the *tdh* and *trh* genes and the pathogenicity of *V. parahaemolyticus* remains unclear. In the present study, 13 isolates from aquatic products (VPF-05, 10, 12, 38, 56, 80, 81, 90, 93, 102, 106, 128, and 130, Table 1) were positive for the *tdh* gene. Moreover, some of the *V. parahaemolyticus* isolates did not contain *tdh* or *trh*, but still remained pathogenic; some environmental isolates lacking *tdh* and/or *trh* have been shown to produce putative virulence factors (Xie *et al.*, 2016). We also found that subgroups C4–C7 (clinical isolates) were clustered with VPF, which lacked the *tdh* and *trh* genes (Fig. 2). Subgroup C5, which harbors three different virulence genes, is a valuable model for research. Horizontal gene transfer may not only provide a route for the spread of virulence factors among *V. parahaemolyticus* strains and steer evolutionary change, but also play a role in the emergence of novel potentially pathogenic strains.

*V. parahaemolyticus* is a human and shrimp pathogen. Previous studies (Kondo *et al.*, 2014; De *et al.*, 2015; Koiwai *et al.*, 2015; Fu *et al.*, 2018) reported that AHPND-causing strains lacked the conventional virulence factors *tdh* and/or *trh* of human pathogenic strains. However, limited information is currently available on the epidemiology of *V. parahaemolyticus* AHPND-causing strains, and the genetic relationship between AHPND-causing strains and other sources of *V. parahaemolyticus* has not yet been elucidated in detail. The microevolutionary relationship between these two strain types from AHPND-infected shrimp and clinical samples were discussed for the first time in the present study. The results obtained on the phylogenetic relationship showed that all VPE strains may be categorized into three small groups with an even distribution on phylogenetic trees, which revealed that the strains are highly diverse. Furthermore, four isolates (VPC-23,27; VPE-01,02) exhibited a very close phylogenetic relationship (Fig. 2), and except for VPE-01 and 02, which do not encode hemolysin genes, the two other isolates possessed at least one of the *tdh* and *trh* genes. During the process of evolution, pathogenic bacteria appeared in strains isolated from aquatic products, which suggests that anthropogenic activities play a role in the process of transforming strains from non-pathogenic to pathogenic bacteria during the aquaculture process. Moreover, anthropogenic activities potentially have an impact on strains that are capable of causing human illness or disease in shrimp, making them more likely to generate intra- or interspecies genetic communication via recombinant or horizontal gene transfer or mutations to some extent.

Heterogeneity is a strategy for microbes to survive in complex and changing environments (Hallet, 2001; Zhang and Orth, 2013). Previous studies demonstrated that factors such as temperature and salt concentrations exert different effects on microbial growth heterogeneity (Miles *et al.*, 1997; Fujikawa *et al.*, 2009; Liu *et al.*, 2016) in the laboratory environment. In the present study, we attempted to elucidate the relationships between growth heterogeneity, external environments (out of the lab), and genetic diversity. In total, 134 *V. parahaemolyticus* isolates (VPF group) and 10 different species of aquatic products were categorized into two sources (freshwater and seawater), and we observed the SNPs of 7 housekeeping gene fragments in each strain (Supplementary Table S2). The average numbers of SNPs in the freshwater and seawater sources were similar; however, a significant difference was observed between species isolated from aquatic products. In Supplementary Table S2, the highest AVG number of SNPs was observed in *Palaemon modestus* (Prawn) from freshwater (55.00), while the lowest was in *Penaeus vannamei* (Shrimp) from seawater (46.03). This result indicates that the diversity of *V. parahaemolyticus* exerts a greater impact at the species level than that due to living conditions in non-lab environments. However, some external factors, such as stress conditions, strain density, or anthropogenic activities, may have influenced the rate of generation of genetic diversity. Phenotypic differences and inheritance variations are used by microbes to adapt to changes in the living environment. Furthermore, under environmental selective pressure, the heterogeneity observed among different isolated *V. parahaemolyticus* strains may play an important role in the generation of genetic diversity mechanisms.

## Conclusion

The relationships between virulence factors, genetic diversity, and the SNPs of *V. parahaemolyticus* isolated from clinical and seafood sources in Shanghai and AHPND-infected shrimp in Guangdong province were discussed herein for the first time. Consistent with the MLST scheme, novel allelic profiles and STs indicate the high genetic diversity of these isolated *V. parahaemolyticus* strains. Furthermore, the strains that cause human illness or disease in shrimps are more likely to generate intra- or inter-species genetic communication by generating recombinant or horizontal gene transfer or mutations. Phylogenetic analyses indicated that some of the clinical isolates closely correlated with the isolates recovered from AHPND-infected shrimp and other environmental sources. These results further confirm that anthropogenic activities and environmental selective pressure affect strains potentially capable of causing illness in humans or disease in shrimp and generate intra- or interspecies gene communication via recombinant or horizontal gene transfer or causing mutations to some extent.

## Citation

Lu, Y., Yang, L., Meng, J., Zhao, Y., Song, Y., Zhu, Y., et al. (2020) Microevolution of *Vibrio parahaemolyticus* Isolated from Clinical, Acute Hepatopancreatic Necrosis Disease Infecting Shrimps, and Aquatic Production in China. *Microbes Environ ***35**: ME19095.

https://doi.org/10.1264/jsme2.ME19095

## Supplementary Material

Supplementary Material

## Figures and Tables

**Table 1.** Properties of 184 *V. parahaemolyticus* isolates

Isolate	Source	Year	*tlh*^a^	*tdh*^a^	*trh*^a^	ST	Allelic profile (*dna*E, *gyr*B, *rec*A, *dtd*S, *pnt*A, *pyr*C, *tna*A)^b^
VPF-01	Macrobrachium nipponense (Prawn)	April 2014	√	×	×	1953*	5, 304, 61, 19, 18, 3, 90
VPF-02	Macrobrachium nipponense (Prawn)	April 2014	√	×	×	1043	26, 220, 168, 47, 11, 168, 122
VPF-03	Macrobrachium nipponense (Prawn)	April 2014	√	×	×	362	36, 194, 44, 67, 102, 5, 37
VPF-04	Macrobrachium nipponense (Prawn)	April 2014	√	×	×	1954*	5, 106, 89, 61, 4, 37, 24
VPF-05	Macrobrachium nipponense (Prawn)	April 2014	√	√	×	1980*	42, 399, 244, 349, 23, 233, 24
VPF-06	Macrobrachium nipponense (Prawn)	April 2014	√	×	×	1980*	42, 399, 244, 349, 23, 233, 24
VPF-07	Macrobrachium nipponense (Prawn)	April 2014	√	×	×	1980*	42, 399, 244, 349, 23, 233, 24
VPF-08	Macrobrachium nipponense (Prawn)	April 2014	√	×	×	864	3, 230, 61, 268, 2, 245, 2
VPF-09	Macrobrachium nipponense (Prawn)	April 2014	√	×	×	2000*	133, 480, 62, 306, 31, 5, 33
VPF-10	Macrobrachium nipponense (Prawn)	April 2014	√	√	×	2000*	133, 480, 62, 306, 31, 5, 33
VPF-11	Macrobrachium nipponense (Prawn)	May 2014	√	×	×	284	135, 168, 98, 140, 56, 11, 33
VPF-12	Macrobrachium nipponense (Prawn)	May 2014	√	√	×	1823*	112, 4, 77, 92, 60, 8, 26
VPF-13	Macrobrachium nipponense (Prawn)	May 2014	√	×	×	284	135, 168, 98, 140, 56, 11, 33
VPF-14	Macrobrachium nipponense (Prawn)	May 2014	√	×	×	284	135, 168, 98, 140, 56, 11, 33
VPF-15	Macrobrachium nipponense (Prawn)	May 2014	√	×	×	284	135, 168, 98, 140, 56, 11, 33
VPF-16	Macrobrachium nipponense (Prawn)	May 2014	√	×	×	1991*	80, 261, 25, 185, 6, 74, 33
VPF-17	Macrobrachium nipponense (Prawn)	May 2014	√	×	×	284	135, 186, 98, 140, 56, 11, 33
VPF-18	Macrobrachium nipponense (Prawn)	May 2014	√	×	×	1823*	112, 4, 77, 92, 60, 8, 26
VPF-19	Macrobrachium nipponense (Prawn)	May 2014	√	×	×	284	135, 168, 98, 140, 56, 11, 33
VPF-20	Macrobrachium: Palaemonidae (Prawn)	July 2014	√	×	×	799	28, 4, 82, 88, 63, 187, 1
VPF-21	Penaeus monodon (Shrimp)	July 2014	√	×	×	1972*	31, 482, 90, 360, 61, 5, 50
VPF-22	Macrobrachium: Palaemonidae (Prawn)	July 2014	√	×	×	1823*	112, 4, 77, 92, 60, 8, 26
VPF-23	Penaeus monodon (Shrimp)	September 2014	√	×	×	669	203, 152, 224, 69, 18, 11, 26
VPF-24	Penaeus vannamei (Shrimp)	September 2014	√	×	×	408	111, 188, 164, 149, 115, 164, 118
VPF-25	Ostrea gigas tnunb (Seafood)	September 2014	√	×	×	1974*	33, 180, 30, 27, 69, 221, 226
VPF-26	Ostrea gigas tnunb (Seafood)	September 2014	√	×	×	2002*	148, 73, 59, 19, 61, 391, 24
VPF-27	Clam (Seafood)	September 2014	√	×	×	1353	190, 15, 31, 55, 18, 58, 23
VPF-28	razor Clam (Seafood)	September 2014	√	×	×	1966*	26, 71, 31, 13, 26, 184, 12
VPF-29	Macrobrachium nipponense (Prawn)	September 2014	√	×	×	1955*	5, 4, 98, 112, 21, 41, 47
VPF-30	Penaeus monodon (Shrimp)	September 2014	√	×	×	1935*	188, 271, 62, 151, 2, 37, 9
VPF-31	Penaeus vannamei (Shrimp)	September 2014	√	×	×	415	42, 134, 99, 79, 26, 41, 51
VPF-32	Penaeus vannamei (Shrimp)	September 2014	√	×	×	1955*	5, 4, 98, 112, 21, 41, 47
VPF-33	Ostrea gigas tnunb (Seafood)	September 2014	√	×	√	1956*	5, 159, 36, 35, 37, 46, 23
VPF-34	Clam (Seafood)	September 2014	√	×	×	1963*	14, 30, 141, 78, 4, 11, 76
VPF-35	razor Clam (Seafood)	September 2014	√	×	×	2005*	159, 236, 31, 342, 26, 296, 58
VPF-36	Scallop (Seafood)	September 2014	√	×	×	839	248, 104, 67, 306, 28, 78, 198
VPF-37	Macrobrachium: Palaemonidae (Prawn)	October 2014	√	×	×	376	160, 203, 15, 118, 82, 5, 80
VPF-38	Macrobrachium: Palaemonidae (Prawn)	October 2014	√	√	×	234	5, 84, 115, 74, 84, 159, 84
VPF-39	Penaeus monodon (Shrimp)	October 2014	√	×	×	1968*	28, 106, 310, 19, 26, 5, 94
VPF-40	Clam (Seafood)	October 2014	√	×	×	863	2, 198, 72, 94, 26, 7, 94
VPF-41	Penaeus monodon (Shrimp)	October 2014	√	×	×	1938*	254, 8, 17, 275, 2, 122, 26
VPF-42	Macrobrachium: Palaemonidae (Prawn)	October 2014	√	×	×	1976*	35, 130, 239, 27, 18, 45, 12
VPF-43	Penaeus monodon (Shrimp)	October 2014	√	×	×	1957*	5, 84, 115, 74, 84, 159, 46
VPF-44	Penaeus monodon (Shrimp)	October 2014	√	×	×	1969*	28, 15, 31, 55, 18, 58, 46
VPF-45	Ostrea gigas tnunb (Seafood)	October 2014	√	×	×	1977*	35, 104, 61, 353, 19, 121, 13
VPF-46	Clam (Seafood)	October 2014	√	×	×	1689	179, 232, 351, 415, 125, 5, 26
VPF-47	razor Clam (Seafood)	October 2014	√	×	×	1993*	84, 195, 89, 19, 26, 10, 26
VPF-48	Scallop (Seafood)	October 2014	√	×	×	1975*	33, 87, 24, 5, 10, 82, 1
VPF-49	Salmon (Seafood)	October 2014	√	×	×	1964*	14, 184, 188, 218, 28, 82, 51
VPF-50	Macrobrachium: Palaemonidae (Prawn)	November 2014	√	×	×	415	42, 134, 99, 79, 26, 41, 51
VPF-51	Macrobrachium: Palaemonidae (Prawn)	November 2014	√	×	×	415	42, 134, 99, 79, 26, 41, 51
VPF-52	Macrobrachium nipponense (Prawn)	November 2014	√	×	×	1937*	251, 4, 220, 69, 50, 296, 23
VPF-53	Macrobrachium nipponense (Prawn)	November 2014	√	×	×	1476	104, 349, 25, 282, 26, 141, 51
VPF-54	Macrobrachium: Palaemonidae (Prawn)	November 2014	√	×	×	1942*	356, 438, 346, 27, 26, 104, 54
VPF-55	Macrobrachium: Palaemonidae (Prawn)	November 2014	√	×	×	832	28, 349, 102, 284, 46, 141, 26
VPF-56	Macrobrachium nipponense (Prawn)	November 2014	√	√	×	3	3, 4, 19, 4, 29, 4, 22
VPF-57	Macrobrachium nipponense (Prawn)	November 2014	√	×	×	832	28, 349, 102, 284, 26, 141, 26
VPF-58	Macrobrachium: Palaemonidae (Prawn)	November 2014	√	×	×	411	2, 113, 72, 94, 26, 83, 23
VPF-59	Macrobrachium: Palaemonidae (Prawn)	November 2014	√	×	×	1933*	169, 191, 151, 73, 26, 46, 94
VPF-60	Penaeus monodon (Shrimp)	December 2014	√	×	×	1995*	100, 122, 31, 69, 47, 333, 99
VPF-61	Penaeus vannamei (Shrimp)	December 2014	√	×	√	1925*	234, 285, 74, 278, 61, 78, 57
VPF-62	Ostrea gigas tnunb (Seafood)	December 2014	√	×	×	1978*	35, 280, 11, 90, 23, 171, 24
VPF-63	razor Clam (Seafood)	December 2014	√	×	×	1986*	47, 139, 53, 19, 3, 143, 26
VPF-64	Macrobrachium nipponense (Prawn)	December 2014	√	×	×	1946*	3, 379, 269, 13, 192, 46, 33
VPF-65	Macrobrachium: Palaemonidae (Prawn)	December 2014	√	×	√	2001*	138, 466, 207, 19, 26, 377, 23
VPF-66	Penaeus monodon (Shrimp)	December 2014	√	×	√	1995*	100, 122, 31, 69, 47, 333, 99
VPF-67	Penaeus monodon (Shrimp)	December 2014	√	×	×	355	153, 191, 70, 19, 23, 8, 1
VPF-68	Penaeus vannamei (Shrimp)	December 2014	√	×	×	1823	112, 4, 77, 92, 60, 8, 26
VPF-69	razor Clam (Seafood)	December 2014	√	×	×	248	5, 88, 61, 19, 18, 3, 90
VPF-70	razor Clam (Seafood)	December 2014	√	×	√	248	5, 88, 61, 19, 18, 3, 90
VPF-71	Macrobrachium: Palaemonidae (Prawn)	December 2014	√	×	×	1160*	153, 191, 70, 19, 6, 8, 1
VPF-72	Penaeus vannamei (Shrimp)	December 2014	√	×	√	1160*	153, 191, 70, 19, 6, 8, 1
VPF-73	Ostrea gigas tnunb (Seafood)	December 2014	√	×	×	1981*	42, 419, 30, 290, 55, 266, 23
VPF-74	Palaemon modestus (Prawn)	December 2014	√	×	×	338	149, 184, 31, 76, 98, 11, 84
VPF-75	Penaeus monodon (Shrimp)	January 2015	√	×	×	1985*	44, 2, 61, 69, 4, 373, 23
VPF-76	Penaeus monodon (Shrimp)	January 2015	√	×	×	1996*	102, 212, 91, 169, 23, 5, 26
VPF-77	Penaeus vannamei (Shrimp)	January 2015	√	×	×	1934*	171, 222, 113, 69, 4, 5, 26
VPF-78	Macrobrachium nipponense (Prawn)	February 2015	√	×	×	428	171, 222, 113, 126, 4, 62, 23
VPF-79	Penaeus vannamei (Shrimp)	February 2015	√	×	×	392	5, 84, 115, 74, 63, 159, 84
VPF-80	Macrobrachium nipponense (Prawn)	February 2015	√	√	×	1970*	28, 144, 116, 138, 219, 177, 81
VPF-81	Macrobrachium nipponense (Prawn)	March 2015	√	√	×	1988*	51, 43, 97, 13, 46, 46, 57
VPF-82	Penaeus vannamei (Shrimp)	March 2015	√	×	×	1210	270, 414, 194, 13, 205, 5, 57
VPF-83	Macrobrachium nipponense (Prawn)	March 2015	√	×	×	1971*	28, 144, 116, 252, 26, 54, 61
VPF-84	Penaeus vannamei (Shrimp)	March 2015	√	×	×	1946*	3, 379, 269, 13, 192, 46, 33
VPF-85	Macrobrachium nipponense (Prawn)	April 2015	√	×	×	1958*	5, 136, 206, 213, 207, 212, 54
VPF-86	Penaeus vannamei (Shrimp)	April 2015	√	×	×	1806*	215, 344, 218, 258, 183, 232, 17
VPF-87	Penaeus vannamei (Shrimp)	April 2015	√	×	×	1806*	215, 344, 218, 258, 183, 232, 17
VPF-88	Macrobrachium nipponense (Prawn)	April 2015	√	×	×	247	116, 149, 107, 76, 45, 62, 26
VPF-89	Macrobrachium nipponense (Prawn)	April 2015	√	√	×	248	5, 88, 61, 19, 18, 3, 90
VPF-90	Penaeus vannamei (Shrimp)	April 2015	√	×	×	248	5, 88, 61, 19, 18, 3, 90
VPF-91	Penaeus vannamei (Shrimp)	June 2015	√	×	×	411	2, 113, 72, 94, 26, 83, 23
VPF-92	Macrobrachium nipponense (Prawn)	June 2015	√	×	√	1999*	126, 195, 123, 66, 46, 195, 26
VPF-93	Macrobrachium nipponense (Prawn)	June 2015	√	√	×	1939*	270, 371, 273, 13, 69, 238, 26
VPF-94	Penaeus vannamei (Shrimp)	June 2015	√	√	×	1940*	270, 371, 273, 103, 69, 238, 26
VPF-95	Penaeus vannamei (Shrimp)	June 2015	√	×	×	843	167, 4, 152, 103, 107, 153, 47
VPF-96	Macrobrachium nipponense (Prawn)	June 2015	√	×	×	1941*	299, 205, 102, 29, 28, 52, 178
VPF-97	Macrobrachium nipponense (Prawn)	June 2015	√	×	×	411	2, 113, 72, 94, 26, 83, 23
VPF-98	Penaeus vannamei (Shrimp)	June 2015	√	×	×	1932*	167, 147, 67, 206, 56, 37, 23
VPF-99	Penaeus vannamei (Shrimp)	July 2015	√	×	×	408	111, 188, 164, 149, 115, 164, 118
VPF-100	Macrobrachium nipponense (Prawn)	July 2015	√	×	×	121	3, 2, 82, 50, 4, 78, 66
VPF-101	Macrobrachium nipponense (Prawn)	July 2015	√	×	×	1945*	1, 89, 98, 161, 176, 263, 86
VPF-102	Penaeus vannamei (Shrimp)	July 2015	√	√	×	1947*	3, 402, 292, 125, 152, 214, 94
VPF-103	Penaeus vannamei (Shrimp)	July 2015	√	×	×	1960*	11, 117, 123, 244, 50, 130, 119
VPF-104	Penaeus vannamei (Shrimp)	July 2015	√	×	×	1582	299, 4, 286, 222, 2, 184, 79
VPF-105	Macrobrachium nipponense (Prawn)	August 2015	√	×	×	370	19, 196, 149, 171, 105, 11, 23
VPF-106	Penaeus vannamei (Shrimp)	August 2015	√	√	×	1992*	80, 88, 111, 284, 37, 95, 61
VPF-107	Penaeus vannamei (Shrimp)	August 2015	√	×	×	1948*	3, 349, 102, 284, 26, 141, 51
VPF-108	Penaeus vannamei (Shrimp)	August 2015	√	×	×	1998*	118, 428, 123, 278, 21, 11, 51
VPF-109	Penaeus vannamei (Shrimp)	September 2015	√	×	×	1592*	36, 184, 31, 76, 98, 11, 84
VPF-110	Macrobrachium nipponense (Prawn)	September 2015	√	×	×	1959*	5, 136, 206, 180, 207, 212, 54
VPF-111	Macrobrachium nipponense (Prawn)	September 2015	√	×	×	490	118, 253, 72, 76, 50, 184, 54
VPF-112	Penaeus vannamei (Shrimp)	September 2015	√	√	×	1990*	73, 159, 67, 212, 31, 89, 26
VPF-113	Macrobrachium nipponense (Prawn)	September 2015	√	×	×	12	9, 21, 15, 13, 4, 10, 26
VPF-114	Penaeus vannamei (Shrimp)	September 2015	√	×	×	1949*	3, 403, 227, 353, 182, 11, 66
VPF-115	Penaeus vannamei (Shrimp)	October 2015	√	×	×	503	19, 226, 61, 207, 131, 11, 132
VPF-116	Macrobrachium nipponense (Prawn)	October 2015	√	×	×	1967*	27, 171, 25, 112, 28, 181, 23
VPF-117	Macrobrachium nipponense (Prawn)	October 2015	√	×	×	1987*	47, 287, 19, 29, 28, 18, 51
VPF-118	Penaeus vannamei (Shrimp)	October 2015	√	×	×	236	114, 100, 61, 122, 66, 54, 85
VPF-119	Penaeus vannamei (Shrimp)	October 2015	√	×	×	1961*	11, 283, 113, 201, 50, 47, 157
VPF-120	Macrobrachium nipponense (Prawn)	October 2015	√	×	×	2004*	153, 191, 86, 19, 6, 175, 51
VPF-121	Penaeus vannamei (Shrimp)	October 2015	√	×	×	1936*	233, 165, 242, 69, 41, 175, 51
VPF-122	Penaeus vannamei (Shrimp)	November 2015	√	×	×	415	42, 134, 99, 79, 26, 41, 51
VPF-123	Penaeus vannamei (Shrimp)	November 2015	√	×	×	1982*	42, 22, 99, 79, 26, 41, 73
VPF-124	Macrobrachium nipponense (Prawn)	November 2015	√	×	×	471	175, 22, 168, 201, 130, 17, 73
VPF-125	Macrobrachium nipponense (Prawn)	November 2015	√	×	×	1997*	104, 349, 25, 282, 26, 141, 51
VPF-126	Penaeus vannamei (Shrimp)	November 2015	√	×	×	1994*	98, 4, 136, 107, 77, 8, 23
VPF-127	Penaeus vannamei (Shrimp)	November 2015	√	×	×	832	28, 349, 102, 284, 26, 141, 26
VPF-128	Macrobrachium nipponense (Prawn)	November 2015	√	√	×	3	3, 4, 19, 4, 29, 4, 22
VPF-129	Penaeus vannamei (Shrimp)	November 2015	√	×	×	832	28, 349, 102, 284, 26, 141, 26
VPF-130	Penaeus vannamei (Shrimp)	November 2015	√	√	×	395	169, 104, 151, 73, 26, 46, 94
VPF-131	Penaeus vannamei (Shrimp)	December 2015	√	×	×	1983*	42, 330, 3, 297, 26, 109, 31
VPF-132	Macrobrachium nipponense (Prawn)	December 2015	√	×	×	2003*	149, 130, 98, 76, 98, 11, 84
VPF-133	Penaeus vannamei (Shrimp)	December 2015	√	×	×	1979*	35, 130, 118, 372, 193, 128, 24
VPF-134	Penaeus vannamei (Shrimp)	December 2015	√	×	×	1973*	31, 153, 135, 74, 19, 128, 238
VPC-01	Clinical	April 2015	√	√	×	3	3, 4, 19, 4, 29, 4, 22
VPC-02	Clinical	April 2015	√	√	×	3	3, 4, 19, 4, 29, 4, 22
VPC-03	Clinical	April 2015	√	√	×	3	3, 4, 19, 4, 29, 4, 22
VPC-04	Clinical	May 2015	√	√	×	1950*	3, 331, 19, 4, 29, 4, 22
VPC-05	Clinical	May 2015	√	√	×	3	3, 4, 19, 4, 29, 4, 22
VPC-06	Clinical	May 2015	√	×	√	1130*	5, 52, 27, 13, 46, 330, 77
VPC-07	Clinical	May 2015	√	√	×	1130*	5, 52, 27, 13, 46, 330, 77
VPC-08	Clinical	May 2015	√	√	×	3	3, 4, 19, 4, 29, 4, 22
VPC-09	Clinical	September 2015	√	×	√	332	14, 30, 141, 78, 4, 37, 13
VPC-10	Clinical	September 2015	√	√	×	345	11, 48, 19, 48, 26, 48, 26
VPC-11	Clinical	September 2015	√	√	×	216	98, 135, 112, 48, 77, 97, 26
VPC-12	Clinical	October 2015	√	√	×	3	3, 4, 19, 4, 29, 4, 22
VPC-13	Clinical	October 2015	√	√	×	3	3, 4, 19, 4, 29, 4, 22
VPC-14	Clinical	October 2015	√	√	×	332	14, 30, 141, 78, 4, 37, 13
VPC-15	Clinical	October 2015	√	√	×	3	3, 4, 19, 4, 29, 4, 22
VPC-16	Clinical	November 2015	√	√	×	345	11, 48, 19, 48, 26, 48, 26
VPC-17	Clinical	November 2015	√	√	×	3	3, 4, 19, 4, 29, 4, 22
VPC-18	Clinical	December 2015	√	√	√	3	3, 4, 19, 4, 29, 4, 22
VPC-19	Clinical	January 2016	√	×	√	1962*	11, 4, 15, 150, 23, 298, 1
VPC-20	Clinical	March 2016	√	√	×	332	14, 30, 141, 78, 4, 37, 13
VPC-21	Clinical	March 2016	√	√	×	1965*	14, 419, 141, 78, 82, 37, 13
VPC-22	Clinical	April 2016	√	√	×	345	11, 48, 19, 107, 26, 48, 26
VPC-23	Clinical	June 2016	√	√	√	813	33, 261, 93, 151, 176, 52, 194
VPC-24	Clinical	June 2016	√	√	×	1952*	3, 4, 19, 151, 29, 4, 22
VPC-25	Clinical	June 2016	√	√	×	1951*	3, 4, 19, 4, 93, 4, 22
VPC-26	Clinical	July 2016	√	√	×	331	147, 181, 127, 69, 26, 4, 23
VPC-27	Clinical	July 2016	√	×	√	332	14, 30, 141, 78, 4, 37, 13
VPC-28	Clinical	August 2016	√	√	×	1463*	309, 111, 167, 188, 116, 355, 33
VPC-29	Clinical	August 2016	√	√	×	3	3, 4, 19, 4, 29, 4, 22
VPC-30	Clinical	August 2016	√	√	×	3	3, 4, 19, 4, 29, 4, 22
VPC-31	Clinical	September 2016	√	√	×	332	14, 30, 141, 78, 4, 37, 13
VPC-32	Clinical	September 2016	√	√	×	3	3, 4, 19, 4, 29, 4, 22
VPC-33	Clinical	September 2016	√	√	×	3	3, 4, 19, 4, 29, 4, 22
VPC-34	Clinical	September 2016	√	×	×	199	22, 28, 17, 13, 8, 19, 14
VPC-35	Clinical	September 2016	√	√	×	1467*	3, 40, 36, 41, 114, 39, 26
VPC-36	Clinical	October 2016	√	√	×	3	3, 4, 19, 4, 29, 4, 22
VPC-37	Clinical	October 2016	√	√	×	654	35, 110, 29, 78, 10, 86, 51
VPC-38	Clinical	November 2016	√	√	×	345	11, 48, 19, 48, 26, 48, 26
VPC-39	Clinical	November 2016	√	√	×	3	3, 4, 19, 4, 29, 4, 22
VPC-40	Clinical	December 2016	√	√	√	332	14, 30, 141, 78, 4, 37, 13
VPE-01	AHPND	June 2015	√	×	×	1061	183, 396, 172, 27, 23, 182, 57
VPE-02	AHPND	June 2015	√	×	×	1061	183, 396, 172, 27, 23, 182, 57
VPE-03	AHPND	June 2015	√	×	×	1984*	42, 134, 172, 79, 26, 41, 51
VPE-04	AHPND	July 2015	√	×	×	1984*	42, 134, 172, 79, 26, 41, 51
VPE-05	AHPND	July 2015	√	×	×	415	42, 134, 99, 79, 26, 41, 51
VPE-06	AHPND	August 2015	√	×	×	114	55, 15, 31, 55, 18, 58, 46
VPE-07	AHPND	August 2015	√	×	×	114	55, 15, 31, 55, 18, 58, 46
VPE-08	AHPND	August 2015	√	×	×	1989*	55, 15, 86, 55, 18, 58, 46
VPE-09	AHPND	September 2015	√	×	×	114	55, 15, 31, 55, 18, 58, 46
VPE-10	AHPND	September 2015	√	×	×	114	55, 15, 31, 55, 18, 58, 46

^a^ √, present; ×, absent. ^b^ The numbers in this column represent the allele designation at each locus. * Novel STs
