# Supplementary material for: Microevolution of Vibrio parahaemolyticus Isolated from Clinical, Acute Hepatopancreatic Necrosis Disease Infecting Shrimps, and Aquatic Production in China
Source: Microbes Environ. 2020 Mar 20;35(2):ME19095. doi: 10.1264/jsme2.ME19095 (PMC7308574; doi:10.1264/jsme2.ME19095)
Supplement: Supplementary file 1 — Supplementary Material [file 35_19095_s1.pdf]

TABLE S1 Sequence of primers used in multilocus sequence typing

Primer	Gene	Sequence of primers (5–3’)	Amplification length (bp)
*dna*E-F	*dna*E	CGRATMACCGCTTTCGCCG	596
*dna*E-R		GAKATGTGTGAGCTGTTTGC	
gyrB-F	*gyrB*	GAAGGBGGTATTCAAGC	629
gyrB-R		GAGTCACCCTCCACWATGTA	
recA-F	*recA*	GAAACCATTTCAACGGGTTC	773
recA-F		CCATTGTAGCTGTACCAAGCACCC	
dtdS-F	*dtdS*	TGG CCA TAA CGA CAT TCT GA	497
dtdS-R		GAG CAC CAA CGT GTT TAG C	
pntA-F	*pntA*	ACGGCTACGCAAAAGAAATG	470
pntA-R		TTGAGGCTGAGCCGATACTT	
pyrC-F	*pyrC*	AGCAACCGGTAAAATTGTCG	553
pyrC-R		CAGTGTAAGAACCGGCACAA	
tnaA-F	*tnaA*	TGTACGAAATTGCCACCAAA	463
tnaA-R		AATATTTTCGCCGCATCAAC	

TABLE S2 Nucleotide diversity of MLST loci for 134 *V. parahaemolyticus* isolates

Source	No. of SNPs(*dnaE, gyrB, recA, dtdS, pntA, pyrC, tnaA*)	Sum of SNPs	No. of Samples	AVG of SNPs
Macrobranchium nipponense(Prawn)	300, 445, 516, 421, 129, 334, 254	2399	49	48.96
Macrobrachium Palaemonidae(Prawn)	75, 106, 191, 123, 25, 103, 59	682	13	52.46
Palaemum modeslus(Prawn)	6, 6, 19, 12, 2, 6, 4	55	1	55.00
Penaeus monodon(Shrimp)	70, 93, 175, 86, 27, 79, 56	586	12	48.83
Penaeus vannamei(Shrimp)	243, 365, 309, 356, 111, 256, 201	1841	40	46.03
Ostreagigastnunb(Seafood)	32, 47, 85, 56, 17, 48, 40	325	6	54.17
Clam(Seafood)	26, 25, 74, 36, 6, 25, 25	217	4	54.25
razor Clam(Seafood)	31, 45, 108, 38, 6, 48, 36	312	6	52.00
Scallop(Seafood)	14, 17, 23, 21, 6, 14, 8	103	2	51.50
Salmon(Seafood)	6, 6, 12, 13, 2, 9, 3	51	1	51.00
freshwater	381, 557, 726, 556, 156, 443, 317	3136	63	49.78
seawater	422, 598, 786, 606, 175, 479, 369	3435	71	48.38

Freshwater: Macrobranchium nipponense(Prawn), Macrobranchium nipponense(Prawn), Palaemum modeslus(Prawn)

Seawater: Penaeus monodon(Shrimp), Penaeus vannamei(Shrimp), Ostreagigastnunb(Seafood), Clam(Seafood), Razor Clam(Seafood), Scallop(Seafood), Salmon(Seafood)

TABLE S3 Abbreviations

Abbreviations	Term
AHPND	Acute hepatopancreatic necrosis disease
CC(s)	Clone complexe(s)
EMS	Early mortality syndrome
PCR	Polymerase chain reaction
ST(s)	Sequence type(s)
TCBS	Thiosulfate-citrate-bile salts-sucrose agar culture medium
TDH	Thermostable direct hemolysin
TRH	TDH-related hemolysin
TSB	Tryptic soy broth
VPC	Clinical isolates
VPE	AHPND isolates
VPF	Seafood isolates

TABLE S4 PCR assay

Reaction process		Time	Temperature
Initial denaturation step		3 min	94 ℃
Denaturation		3 min	94 ℃
Annealing*	×25	1 min	62-66 ℃
Extension		2 min	72 ℃
Final extension		3 min	72 ℃

* Annealing temperature of：*tnaA*(62 ℃); *recA, dtdS, gyrB, pyrC, pntA*(64 ℃); *dnaE*(66 ℃).
